# Technical Assistance From State Health Departments for Communities Engaged in Policy, Systems, and Environmental Change: The ACHIEVE Program

**DOI:** 10.5888/pcd10.130093

**Published:** 2013-10-24

**Authors:** Jenny Hefelfinger, Alice Patty, Ann Ussery, Walter Young

**Affiliations:** Author Affiliations: Jenny Hefelfinger, Alice Patty, Ann Ussery, National Association of Chronic Disease Directors, Washington, DC.

## Abstract

**Introduction:**

This study assessed the value of technical assistance provided by state health department expert advisors and by the staff of the National Association of Chronic Disease Directors (NACDD) to community groups that participated in the Action Communities for Health, Innovation, and Environmental Change (ACHIEVE) Program, a CDC-funded health promotion program.

**Methods:**

We analyzed quantitative and qualitative data reported by community project coordinators to assess the nature and value of technical assistance provided by expert advisors and NACDD staff and the usefulness of ACHIEVE resources in the development and implementation of community action plans. A grounded theory approach was used to analyze and categorize phrases in text data provided by community coordinators. Open coding placed conceptual labels on text phrases. Frequency distributions of the quantitative data are described and discussed.

**Results:**

The most valuable technical assistance and program support resources were those determined to be in the interpersonal domain (ie, interactions with state expert advisors, NACDD staff, and peer-to-peer support). The most valuable technical assistance events were action institutes, coaches’ meetings, webinars, and technical assistance conference calls.

**Conclusion:**

This analysis suggests that ACHIEVE communities valued the management and training assistance provided by expert advisors and NACDD staff. State health department expert advisors provided technical guidance and support, including such skills or knowledge-based services as best-practice strategies, review and discussion of community assessment data, sustainability planning, and identification of possible funding opportunities. NACDD staff led development and implementation of technical assistance events.

## Introduction

Action Communities for Health, Innovation, and Environmental Change (ACHIEVE), a federally funded community health promotion initiative, used a collaborative development model to help communities prevent chronic diseases and decrease related risk factors. ACHIEVE was a partnership between local community organizations and national and state organizations whose goal was to create healthy, sustained communities where access to physical activity and healthful foods is high and exposure to tobacco is minimized. ACHIEVE educated and provided technical assistance to community leaders from various health-related or interested organizations on local policy and system and environmental improvements. We used reports submitted by ACHIEVE coaches (the community project coordinators) to analyze the types and value of the technical assistance and support provided by state health department expert and other resources available to ACHIEVE communities, as reported by 33 community project coordinators from 2008 through 2010.

The National Association of Chronic Disease Directors (NACDD) was 1 of 5 national lead organizations that participated in offering technical assistance to additional ACHIEVE communities (1). The other organizations were the National Association of County and City Health Officials (NACCHO), the National Recreation and Park Association (NRPA), the YMCA of the USA (Y-USA), and the Society for Public Health Education (SOPHE). NACDD and these ACHIEVE lead organizations also contracted with outside organizations and individuals to provide program development and specialized technical assistance and training opportunities.

NACDD was the only lead organization that required applicant communities to maintain a formal working relationship with their state health department’s chronic disease program. Consequently, in addition to NACDD staff support, state-based technical assistance was provided by expert advisors. By building relationships between local programs and state health departments, we intended to provide access to state-supported resources, ensure integration with state health department plans, and contribute to the sustainability of local ACHIEVE programs.

The ACHIEVE Program mission was based on the finding that citizen participation in policy development and decision making is an essential element of sustainability and, more specifically, that requiring collaborative partnerships will increase the likelihood that ACHIEVE programs and the local coalitions that manage them will be sustained after ACHIEVE funding has ended (2). The principal goal of ACHIEVE was to develop and implement population-based, risk-focused strategies for physical activity, nutrition, and tobacco use that help prevent or manage health risk factors for heart disease, stroke, diabetes, cancer, obesity, and arthritis. Community health action response teams (CHARTs) included 24 local and county health departments, 7 hospitals or medical centers, 2 nonprofit organizations, and 2 local health-related community coalitions. Participating community members organized to implement policy, systems, and environmental improvements; addressed barriers to physical activity, nutrition, and healthful eating; and reduced tobacco use. These groups recruited and organized local support and resources.

Each CHART provided 2 people called coaches to represent the team and participate in a facilitated leadership model; both coaches had equal responsibility for implementing this project in their community. Coaches typically represented the lead fiscal agency and a local partner agency. CHART members were local community members who represented broad and diverse sectors of the community: schools, universities, health care organizations (including providers and insurers), worksites and businesses, government entities, local organizations and foundations, media, and community planners. These representatives engaged their communities and set priorities for strategic action by developing community action plans (CAPs) to guide and direct their efforts. These plans gave each CHART a road map for building long-term goals (at least 3 years) and short-term objectives (1 year), and for planning activities on a timeline and with a lead partner. CAPs were dynamic documents that enabled adaptations to changes in local priorities, political will, and availability of resources and funding.

State health departments participated in the funding application process with the lead community organization, indicating their commitment to providing sustained technical assistance with data analysis, coalition management, community assessment strategies, and program planning, implementation, and evaluation. Each state health department appointed an expert advisor who offered support and technical assistance to the CHART and coaches. Expert advisors frequently provided expanded support linking communities to other state health department resources and other program technical assistance for media training, funding opportunities, epidemiology, and evaluation support. Expert advisors attended and participated in CHART meetings, webinars, and other required community program components and acted as ex-officio members of the CHART. NACDD staff informed the state’s chronic disease director of emerging program support and resource needs that the expert advisors should fulfill.

NACDD staff consultants provided ongoing support remotely throughout the project funding periods, facilitating technical assistance, providing direct consultation, and referring coaches to resources and content experts. They supported each CHART, helped sustain community activity for CHARTs that were no longer receiving ACHIEVE funding, and helped with the planning and delivery of technical assistance events such as webinars, coaches’ meetings, and action institutes. Throughout 3-year funding cycles, funded coalitions received technical assistance via emails, one-on-one phone calls, conference calls, Web-based support, and site visits. Each community had a primary point of contact with an NACDD consultant.

Staff of the Centers for Disease Control and Prevention (CDC) provided funding, technical support, and guidance from the Healthy Communities Program of the Division of Community Health. They used tools such as the *Sustainability Planning Guide for Healthy Communities* (3) to assist teams with development of sustainability plans and an online success story application to help teams develop stories that were published on the CDC website.

During the first year of ACHIEVE funding, representatives of community coalitions attended 2 in-person coaches’ meetings and action institutes. The meetings included presentations and interactive exercises conducted by nationally recognized experts. These experts provided training and skill-building sessions and tools to assist community coalition members in implementing policy, systems, and environmental change strategies; building effective coalitions; and conducting community assessments.

All ACHIEVE community coaches were provided opportunities to participate in groups called peer learning networks devoted to the process or focus area (physical activity, nutrition, or tobacco) they were working through. These groups had frequent conference calls during which they learned from each other. These interactive community networks were established at national meetings to share program successes and barriers to program development.

In 2010, an online, 6-part social media training component was implemented. Multiple communication platforms including Facebook, Twitter, LinkedIn, YouTube, and Flickr were included. All ACHIEVE communities attended technical assistance and training webinars every 2 months throughout each 3-year contract. Topics included community assessment, evaluation, and community education strategies. Each CHART had access to a private portal that was a secured, password-protected website that allowed them to share program documents, post and access events on a common calendar, manage task lists, and, on a Web forum feature, discuss issues with national partners and community members.

## Methods

We reviewed quantitative and qualitative content reported by community project coordinators on the nature and value of technical assistance provided by expert advisors and NACDD staff and the usefulness of ACHIEVE resources in the development and implementation of community action plans. The quantitative data presented is a compilation of data reported by ACHIEVE local project coordinators in semiannual reports. Because these data included information from 3 separate cohorts (communities funded in 2008, 2009, and 2010), it is aggregated across the 3 years of reporting. The 2008 cohort of communities included 3 years of reports (startup year through the third and final year of funding); the 2009 cohort included 2 years of reports (startup and second year of funding); and the 2010 cohort included 1 year of reports (the startup year). The quantitative variables were selected because they were consistently reported across all 3 NACDD-managed cohorts (2008–2010). ACHIEVE community project coordinators were asked to select responses from a multiple-choice question that asked, “How has your State Expert Advisor been involved in your ACHIEVE activities during the current progress reporting period?” Because semiannual reporting took place over a 3-year period and multiple responses to questions were offered, the frequency with which responses were selected was low. Not all response options were included in the questions during the 3 years of reporting. If the number of respondents is below 61 (Table 1), the question was not asked in each of the 3 years or the answer options were not asked in every community coordinator report. For these reasons, the quantitative data are described and discussed without statistical analyses. The quantitative data are summed across 3 years of semiannual CHART reports. Not all response options were included in the questions during the 3 years of reporting.

**Table 1:** Helpfulness of Resources Provided to ACHIEVE Communities, United States, 2008–2010 (n = 64)

ACHIEVE Resources	Not Helpful, n (%)	Moderately Helpful, n (%)	Very Helpful, n (%)	Not Used, n (%)	Response Count^a^, n
Technical assistance provided by NACDD (eg, conference calls, e-mails, website)	1 (1.6)	15 (23.8)	46 (73.0)	2 (3.2)	64
Webinars and technical assistance conference calls	0	32 (50.8)	28 (44.4)	3 (4.8)	63
Peer-to-peer support (eg, connecting with other communities)	1 (1.6)	24 (38.1)	21 (33.3)	17 (27.0)	63
State health department expert advisors	5 (7.9)	22 (34.9)	30 (47.6)	6 (9.5)	63
State Healthy Communities Coordinator	1 (1.6)	8 (13.1)	20 (32.8)	32 (52.5)	61
Action Institute	0	1 (3.1)	24 (75.0)	7 (21.9)	32
Coaches’ meeting	0	3 (15.8)	15 (78.9)	1 (5.2)	19
ACHIEVE website	1 (10.0)	7 (70.0)	2 (20.0)	0	10
Site visit by NACDD staff	0	1 (7.7)	5 (38.5)	7 (53.8)	13
Technical assistance by CDC or other national partners	1 (10.0)	2 (20.0)	2 (20.0)	5 (50.0)	10

Abbreviations: ACHIEVE, Action Communities for Health, Innovation, and Environmental Change; NACDD, National Association of Chronic Disease Directors; CDC, Centers for Disease Control and Prevention.

a If the number of respondents is below 61, this means the question was not asked in each of the 3 years or the answer options were not asked in every community coordinator report.

One of the quantitative variables reported also included qualitative text information: How were expert advisors involved in ACHIEVE activities? The text data (“other” responses) were analyzed using a grounded theory approach (4) to enrich understanding of the quantitative data. To facilitate analysis, we used open coding to assign codes to each of the 25 narrative responses to this question. Using subjective criteria, directional codes were assigned to each narrative response by the primary author (J.H.), by determining whether the comment reflected a positive, negative, or neutral action on the part of the expert advisors to advance ACHIEVE community initiatives. Codes describing the content of the comment were also assigned. Multiple directional and content codes were assigned to the written responses when the content was multidirectional and reflected more than 1 content area. Quotes from respondents that captured the essence or enriched understanding of the quantitative responses are reported. Assigned codes were reviewed, revised for agreement, and validated by 1 of the co-authors.

## Results

We asked for responses to questions that asked how expert advisors were involved in ACHIEVE activities (Table 2). The most frequent response was to the question, “identifying and sharing best practices on developing policy, systems, and environment strategies” (n = 40; 63%), followed by “providing advice on development of policy, systems, and environmental improvements change strategies” (n = 34; 53%), and “sharing strategies to leverage local government support for ACHIEVE” (n = 27; 42%). The expert advisors were also instrumental in encouraging ACHIEVE communities to disseminate the work they were doing to agencies not funded by ACHIEVE (n = 33; 52%). Half of the reported responses throughout the 3-year reporting period indicated that the “expert advisors participated in local ACHIEVE meetings” (n = 32; 50%). Nearly one-fourth of the responses indicated that the expert advisors “utilized local strategies to inform state policies” (n = 15; 23%). Only 5 responses indicated that the state expert advisors were “not involved” during a reporting period (8%).

**Table 2:** Involvement of State Expert Advisors in ACHIEVE Community Activities, United States, 2008–2010 (n = 64)

Answer Option	Response, n (%)
Identifying and sharing best practices on developing PSE strategies	40 (62.5)
Providing advice on the development of PSE change strategies	34 (53.1)
Offering opportunities for ACHIEVE communities to share with other state agencies	33 (51.6)
Participating in local ACHIEVE meetings	32 (50.0)
Sharing strategies to leverage local government support for ACHIEVE	27 (42.2)
Using local strategies to inform state policies	15 (23.4)
No involvement	5 (7.8)
Other (please describe)	25 (39.0)

Abbreviations: ACHIEVE, Action Communities for Health, Innovation, and Environmental Change; PSE, policy, systems, environment.

Seventeen of the 25 “other” comments (68%) were positive, and the remaining 8 (32%) were neutral. Some of the positive comments were:

. . . we just got a new expert advisor this month. So far, she has been amazing!

In coordination with our City Planner, the expert advisors provided a Complete Streets presentation to the City Planning Commission.

[The expert advisors] looked for funding opportunities within the state.

[Name] was a great sounding board for ideas and thinking processes through.

The neutral comments were observations on the ACHIEVE process, staff turnover, or observations not based on the work of the expert advisors. Some examples of those comments were the following:

Our original expert advisor got a promotion and is no longer working with ACHIEVE.

Our previous state expert advisor left in December.

Our expert advisor provided GoTo Meeting software and technical support to be able to hold CHART meetings and webinars for those unable to travel. . . .

Three of the 25 “other” comments about state expert advisors involvement (12%) were negative (eg, “there has been little assistance from our state expert”).

Five out of 6 (n = 38; 84%) coaches helped collect or analyze community needs assessment data, and 82% (n = 37) reviewed the CAP (Table 3). Other contributions coaches made were presenting assessment data to the CHART or community (n = 32; 71%), discussing assessment data, and drafting the CAP (n = 16 for each activity; 36%). Our results suggest that the most frequent contributions of the CHART executive team and other CHART members were collection or analysis of assessment data and review of the CAP.

**Table 3:** Contributions Made by ACHIEVE Participants to Development of Community Action Plans for ACHIEVE Communities, United States, 2008–2010 (n = 45)

Answer Options	Collected and/or Analyzed Assessment Data	Presented Assessment Data to CHART^a^ or Community	Discussed Assessment Data	Drafted the CAP	Reviewed CAP	Did Not Contribute to CAP

	n (%)					
Coaches	38 (84.4)	32 (71.1)	16 (35.6)	16 (35.6)	37 (82.2)	0
CHART executive team	27 (60.0)	19 (42.2)	10 (22.2)	8 (17.8)	22 (48.9)	0
Other CHART members	33 (73.3)	16 (35.6)	16 (35.6)	10 (22.2)	24 (53.3)	0
Community partner(s)	24 (53.3)	7 (15.6)	10 (22.2)	5 (11.1)	10 (22.2)	0
State health department staff	15 (33.3)	6 (13.3)	11 (24.4)	6 (13.3)	17 (37.8)	1 (2.2)
Other state partner(s)	4 (8.9)	0	2 (4.4)	2 (4.4)	3 (6.6)	4 (8.9)
NACDD staff	9 (20.0)	5 (11.1)	6 (13.3)	7 (15.6)	21 (46.7)	1 (2.2)
Other national partner(s)	2 (4.4)	1 (2.2)	0	0	2 (4.4)	4 (8.9)
CDC staff	5 (11.1)	2 (4.4)	1 (2.2)	0	2 (4.4)	6 (13.3)
Other (describe)	4 (8.9)	0	0	0	1 (2.2)	4 (8.9)

Abbreviations: ACHIEVE, Action Communities for Health, Innovation, and Environmental Change; CHART, Community Health Advisory Resource Team; CAP, community action plan; NACDD, National Association of Chronic Disease Directors; CDC, Centers for Disease Control and Prevention.

a Response counts and percentages do not sum to the total of respondents because not all questions were asked in each report period.

In addition, resources outside of ACHIEVE communities (state health departments and other state partners, staff of NACDD, CDC and other national partners) contributed to the development of the CAP (Table 2). CHART members contributed to the collection and analysis of assessment data (n = 33; 73%). Sixty percent (n = 27) of the CHART executive team contributed to the collection and analysis of assessment data and nearly half (n = 22; 49%) reviewed the CAP. Community partners helped with discussion of assessment data (n = 10; 22%) and review of the CAP (n = 10; 22%). The most frequently mentioned state health department staff contributions were review of the CAP (n = 17; 37.8%) and collection and analysis of assessment data (n = 15; 33.3%). These contributions were followed by discussion of assessment data (n = 11; 24.4%), presentation of assessment data to CHART or community, and drafting of the CAP (n = 6; 13.3% each).

NACDD staff made contributions similar to those made by state health department staff with review of the CAP (n = 21; 46.7%) and collection and analysis of assessment data (n = 9; 20.2%) being the most frequently mentioned contributions. Drafting of the CAP (n = 7; 15.6%), discussion of assessment data (n = 6; 13.3%), and presentation of assessment data to CHART or community (n = 5; 11.1%) were the next most frequent. The most commonly selected contribution from CDC staff was collection and analysis of assessment data with 11.1% of responses (n = 5) indicating this contribution. This low percentage was not unexpected because CDC staff did not have a primary role in providing technical assistance and community project support.

Community coaches reported the helpfulness of technical assistance resources with each report. Coaches meetings were rated as a leading resource in the “very helpful” category (15/19; 78.9%). Three-fourths (24 of 32; 75.0%) of the community coaches rated action institutes as a very helpful resource, followed by the technical assistance provided by NACDD (46 of 63; 73.0%) and the state health department expert advisors (30 of 63; 47.6%). The next most helpful resources were webinars and technical assistance conference calls (28 of 63; 44.4%), site visits by NACDD staff (5 of 13; 38.5%), peer-to-peer support (21 of 63; 33.3%), and State Healthy Communities Coordinators (20 of 61; 32.8%).

## Discussion

State expert advisors were most active in providing technical assistance by identifying best practices and advising ACHIEVE CHARTs on policy, system, and environmental change strategies. This technical assistance supplemented the technical assistance and training that all CHART members received while attending training offerings. Expert advisors assisted communities in understanding and prioritizing assessment data, action planning and implementation, finding ongoing funding to sustain activity, and linking with other healthy community programs.

Because state expert advisors may also have had a broad responsibility to serve other communities in their state, it was perhaps consistent with their job responsibilities that they identified and shared best practices on developing policy, system, and environmental strategies to agencies not funded by ACHIEVE and used local strategies to inform state policies. The low percentage of “no state expert advisor involvement” reported by community coaches suggests that these public health professionals were widely engaged in providing technical assistance. The content of the qualitative text or other data reported supports the high level of active engagement of the expert advisors in ACHIEVE community activity. The analysis of the write-in responses suggests that, at minimum, expert advisors were welcomed by local ACHIEVE coaches, helped present best-practice strategies to community leadership, helped identify funding opportunities for ACHIEVE coalitions, and were responsive to ideas and proposed processes suggested by community coalition members.

Expert advisors were also engaged in the more technical aspects of community coalition activity (eg, helping collect and analyze needs assessment data, reviewing CAPs). Expert advisors may have preferred providing this type of support and coaches frequently acknowledged this support, or both. Expert advisors support that was more technical in nature may have been motivated, at least in part, by the job descriptions at the state health agency, professional interests, or formal training of the expert advisors. Because many community coalition members had no formal public health training, expert advisors may also have recognized a need for a higher level of technical assistance.

The data suggest that coaches made the most significant contributions to the development of CAPs across all communities. Because response frequency for contributions to the development of CAPs for the CHART executive team was comparable to that of other CHART members across all response categories, a broad, cross-section of CHART membership may have contributed at least as much as the executive team. This further suggests that ACHIEVE communities were characterized by their broad, grass-roots community participation. Other national ACHIEVE lead organizations and CDC staff were not expected to provide technical assistance and support to NACDD’s ACHIEVE communities. Consequently, community coordinators reported that these organizations contributed minimal support for the development of NACDD’s ACHIEVE teams community action plans.

The most helpful technical assistance and program support resources to ACHIEVE communities were those provided through interpersonal communications. Face-to-face technical assistance provided by the state expert advisors, NACDD staff, and peer-to-peer interactions was most valued. Other helpful technical assistance events, which also had interpersonal, interactive elements, were the webinars and technical assistance conference calls, the action institutes, and coaches’ meetings.

State health department expert advisors tended to provide technical guidance, including such skills- or knowledge-based services as assistance with best-practice strategies, review and discussion of community assessment data, sustainability planning, and identification of possible funding opportunities. Although regular or day-to-day support provided by expert advisors and NACDD staff may have been valuable for community coordinators (coaches) and their communities, events such as the coaches’ meetings, action institutes, and webinars were complementary learning opportunities that provided intensive, interactive training and education for community coalition members both off- and on-site. These learning events enabled trainers and trainees to ask each other challenging questions. This analysis of community ACHIEVE coach-reported data suggests that community coalitions that engaged in policy, system, and environmental change strategies benefitted greatly from a comprehensive, diverse portfolio of technical assistance and support strategies.
